# Classification and Morphological Parameters of the Scapular Spine: Implications for Surgery

**DOI:** 10.1097/MD.0000000000001986

**Published:** 2015-11-13

**Authors:** Hua-Jun Wang, Hugo Giambini, Da-Biao Hou, Song-Wei Huan, Ning Liu, Jie Yang, Chao Chen, Yan-Ping Gao, Ru-Guo Shang, Yi-Kai Li, Zhen-gang Zha

**Affiliations:** From the First Clinical College, Jinan University and Department of Orthopedics, The First Affiliated Hospital, Jinan University, Guangzhou, China (HJW, DBH, SWH, NL, JY, ZGZ); Biomechanics Laboratory, Division of Orthopedic Research, Mayo Clinic, Rochester, MN, USA (HG); Department of Orthopedics, School of Traditional Chinese Medicine, Southern Medical University, Guangzhou (CC, YKL); Department of Orthopedics, Shenzhen Pingle Orthopedic Hospital, Shenzhen (YPG); and Department of Orthopedics, Guangzhou Orthopedic Hospital, Guangzhou, China (RGS).

## Abstract

Incidence of scapular spine (SS) fractures as a result of complications of reverse total shoulder arthroplasty is relatively high leading to inferior clinical outcomes and an increased risk of revision and dislocation. Fractures of SS because of trauma, including the acromion, constitute 6% to 23% of scapula fractures. The purpose of this study was to classify the SS and present specific geometrical parameters according to osteologic features. A total of 319 intact dry scapulae were collected and classified based on morphological characteristics and shape of the SS. Nine bony landmarks were also chosen and described for their relevance to regions of interest for scapular fixation. Five specific types of SS were noted and the most prevalent groups were Type 1 (Fusiform shape) (47.17%) and Type 5 (Horizontal S-shape) (19.18%). Overall, Types 3, 4, and 1 showed thicker landmark values compared to Type 5, with Type 2 having smaller values. Our classification into 5 distinct types allowed appreciation of the anatomical variance of SSs. The contours of Types 5 and 1 presented a more complex morphology and may lead to a worse surgical approach due to a fracture. As Types 2 and 5 were much thinner than the other types, these may be more susceptible to fractures.

## INTRODUCTION

The scapular spine (SS) is a prominent plate of bone and provides elegant reinforcement to the scapula. It originates from the vertical (scapular) plane of the fossa and attaches to the scapular neck. With the worldwide application of reverse total shoulder arthroplasty, incidence of SS fractures as a result of complications of this procedure is relatively high (0.9%–10%).^1–11^ SS fractures are likely to propagate from a single traumatic event and often find their origin at the tip of the metaglene screw, leading to inferior clinical outcomes and an increased risk of revision and dislocation.^4,5,12,13^ In addition, the SS has been demonstrated to be the most useful region for screw fixation for reverse total shoulder arthroplasty prosthesis on account of more bone stock and cortical thickness which increase screw pullout strength.^14–16^ However, there exists a significant variability in SS morphology, and knowledge of shape and dimension is critically important for these procedures.

Fractures of SS including the acromion constitute 6% to 23% of scapula fractures, approximately the same frequency as glenoid fractures.^17–24^ Direct trauma is mostly seen in high-energy injuries and in combination with other fractures.^17,18^ The cause of indirect trauma is the violent voluntary contraction of muscles and ligaments attached to the SS, often related to stress events with coughing, cuff-tear arthropathy, work-related activities, or sporting activities such as baseball, golf, or football.^19–21^ SS mal-union has been shown to reduce subacromial space and alter normal kinematics of the acromioclavicular and scapulothoracic joints. However, these can be corrected with subacromial opening osteotomy of the SS.^25^ Surgical management for SS fracture is recommended especially in young, fit, and active patients.^17–21^ During these surgical procedures, detailed knowledge of SS is fundamentally important to minimize overlying tissue irritation, aid in fracture reduction, and improve the mechanics of the bone–plate construct.

The SS has been a popular topic involved in surgical management. Quantitative and detailed knowledge of this subject is needed to ensure the best functional outcome without increasing the risk of complications. Until now, the importance of the SS seemed to have been neglected, and few studies have reported quantitative and morphological characteristics of the SS.^14,15,26–28^ Missing anatomical information might have increased hardware removal rate rise to as high as 7.1% because of either implant-related discomfort or failure.^29,30^ In addition, the SS has been versatile used in many areas of the body due to the ease of harvesting, minimal donor site morbidity, as well as the reliable blood supply to this bone.^31–40^ Thus, the purpose of the current study was to classify the SS according to osteologic features on the basis of a large number of Chinese scapulae. In addition, we present specific geometrical parameters and show a comparative analysis between different types and sides of the body which is rarely reported in previous literature and useful for surgical procedures.

## MATERIALS AND METHODS

After ethical approval was obtained from the Human Research Ethics Committee at Southern Medical University, China, a total of 319 intact dry scapulae were obtained from adult specimens from the Department of Anatomy. These consisted on 213 left and 106 right scapulae preserved in hermetic boxes. Age and sex of donors were unknown.

### Classification of the Scapula Spine

**FIGURE 1:**
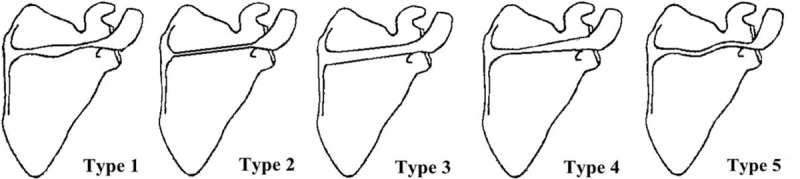
Scapular spine classification schematic: Type 1-Fusiform shape (tapered at both ends and wide in the middle), Type 2-Slender rod shape (thin throughout), Type 3-Thick rod shape (thick throughout), Type 4-Wooden club shape (gradual thickening from medial to lateral edge), and Type 5-Horizontal S-shape (“S” shaped spine).

Two investigators simultaneously classified the scapulae. Classification was based on morphological characteristics, shape, and the course of scapula spine (SS). When any disagreement arose during the observations, a 3rd investigator was consulted for a final determination. Five specific types of SS were noted: Type 1, Fusiform shape (tapered at both ends and wide in the middle); Type 2, Slender rod shape (thin throughout); Type 3, Thick rod shape (thick throughout); Type 4, Wooden club shape (gradual thickening from medial to lateral edge); and Type 5, Horizontal S-shape (“S” shaped spine). Figure 1 shows the different classifications.

### Morphometric Measurements

Morphological features of the SS were observed and measured in all scapulae. Nine bony landmarks/points were chosen for their relevance to regions of interest for scapular fixation and on their measurement reproducibility among specimens. Distances between the points were measured and compared on all left and right SS. Measurements were made using a digital Vernier caliper (Mitutoyo, Japan; accuracy up to 0.01 mm). Thickness at these locations of the spine was measured using a micrometer (Qinghai, China; accuracy up to 0.01 mm). The parameters were as follows:

AE (superior border of SS): length of SS measured from the medial edge of the scapula where it meets with the SS to the lateral edge of the acromion;

BC (lateral border of SS, spinoglenoid notch): height of the spine at the lateral edge;

AC (base border of SS): distance from the medial edge of the scapula where it meets with the SS to the edge of the spinoglenoid notch;

AB: length of SS measured from the medial edge of the scapula where it meets with the SS to point where BC meets with the spine;

AD: length of SS measured from the medial edge of the scapula where it meets with the SS to the corner of the acromion;

FG and HI: height of the spine at point G and I; J, K, L, midpoints of FG, HI, and BC.

**FIGURE 2:**
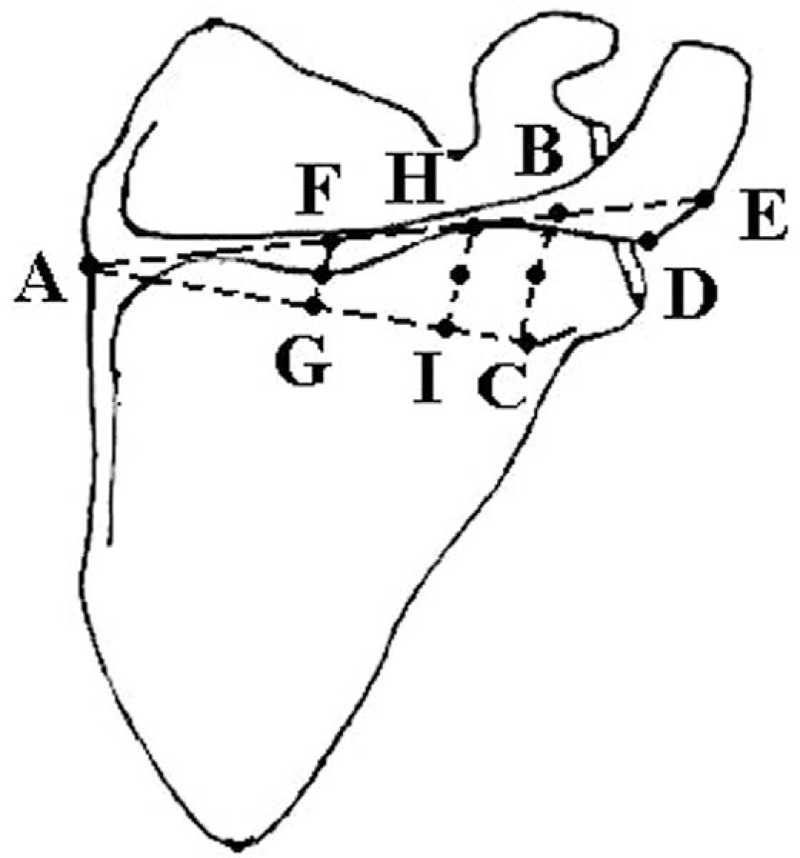
Morphometric measurements: AE (superior border of scapular spine): length of scapular spine measured from the medial edge of the scapula where it meets with the scapular spine to the lateral edge of the acromion; BC (lateral border of scapular spine, spinoglenoid notch): height of the spine at the lateral edge; AC (base border of scapular spine): distance from the medial edge of the scapula where it meets with the scapular spine to the edge of the spinoglenoid notch; AB: length of scapular spine measured from the medial edge of the scapula where it meets with the scapular spine to point where BC meets with the spine; AD: length of scapular spine measured from the medial edge of the scapula where it meets with the scapular spine to the corner of the acromion; FG and HI: height of the spine at point G and I; J, K, L, midpoints of FG, HI, and BC.

Figure 2 describes the location of the bony landmarks. To avoid interobserver variation, all measurements were performed twice by the same author.

### Data Analysis and Statistics

All data are presented as mean and standard deviation (SD). Descriptive statistics was used to describe demographics and measurement variables of all scapulae. Categorical variables are expressed as frequencies and percentages. ANOVA and Student Newman–Keuls were used to compare types considering a *P*-value < 0.05 as statistically significant. The Statistical Package for Social Sciences (SPSS, Chicago, IL; version 13.0) was used for the analysis of the data.

## RESULTS

**FIGURE 3:**
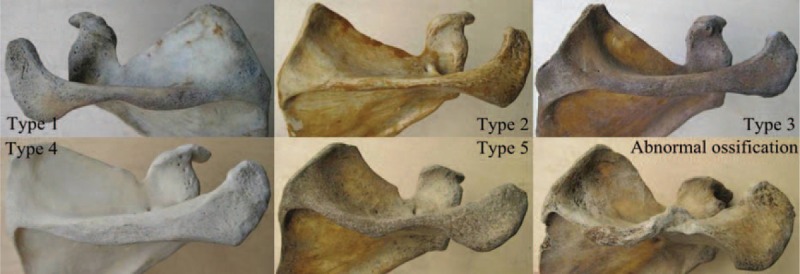
Cadaveric scapular spine classification: Cadaveric scapulae based on their classification are shown together with a scapula presenting a rough surface and abnormal ossification on the crest of the scapular spine. A total of 319 scapulae were obtained and 318 were used to classify into the different types.

Based on morphological classifications, Type 1-Fusiform shape (47.17%) and Type 5-Horizontal S-shape (19.18%) were the most common, followed Type 4-Wooden club shape (13.21%) and Type 3-Thick rod shape (12.58%). Type 2-Slender rod shape (7.86%) was the least common (Table 1). Figure 3 shows the different scapulae types. A unique case presenting a rough surface and abnormal ossification on the crest of the SS was found. This may be due to stress and ossification of the tendon and tendinous fibers of the trapezius muscles. The average length of AE, AC, and BC were 135.83 ± 10.33 mm, 83.27 ± 6.22 mm, and 45.60 ± 5.45 mm, respectively. AD was the shortest and significantly different in Type 2 compared to other types. AB was the shortest for the Type 1 scapulae and significantly different from other types. A complete description and summary of the results can be observed in Table 1. No difference was found between left and right scapulae (Table 2). A summary of the thickness for the bony landmarks on the SS is shown on Table 3. Landmarks B, L, C, H, K, J, and G were thicker in Types 3, 4, and 1 compared to Type 5, with Type 2 being the thinnest. Table 3 shows a complete summary of the measured data. Overall, Types 3, 4, and 1 showed thicker values than Type 5, and Type 2 the thinnest of all cases. No statistical difference was found between left and right sides of the body (Table 4).

**TABLE 1:**
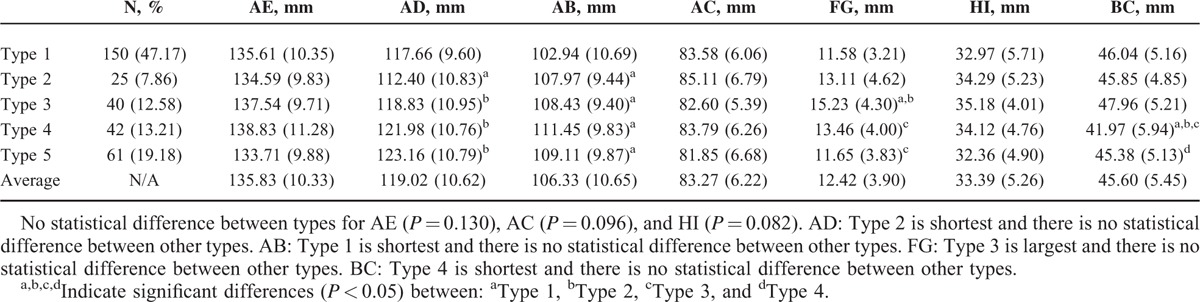
Height, Length Distribution, and Measurements of the Scapular Spine Based on Classification

**TABLE 2:**
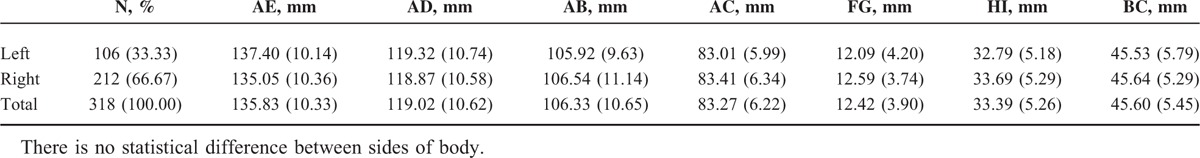
Height and Length Distribution and Measurements of the Scapular Spine Based on Body Side

**TABLE 3:**
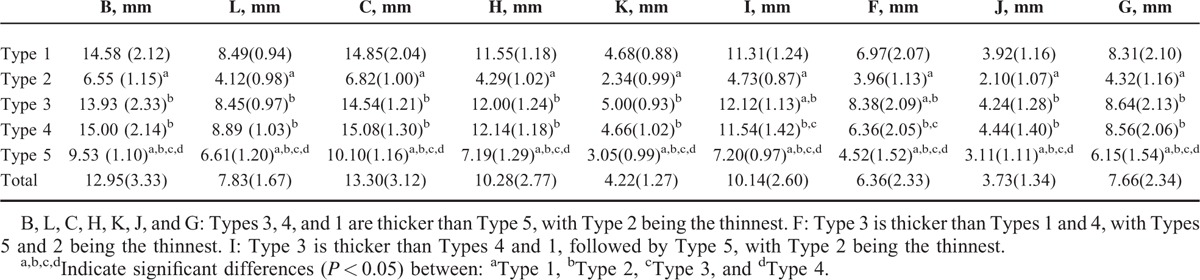
Thickness Distribution and Measurements of the Scapular Spine Based on Classification

**TABLE 4:**
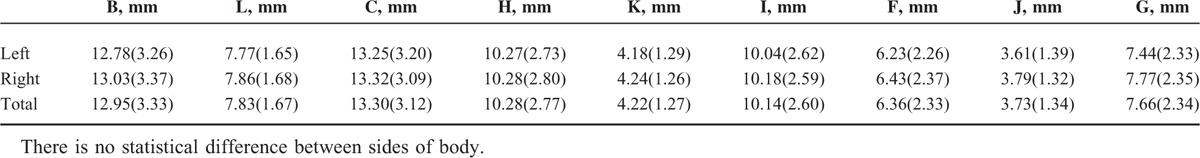
Thickness Distribution and Measurements of the Scapular Spine Based on Body Side

## DISCUSSION

In this study, we successfully classified 318 SSs into 5 types based on their morphological features. Few studies have reported morphological anatomy of SSs, and only 1 of these had a specimen population of 48.^14,15,26,27^ Our results demonstrate that the variation of SS is not a rare occurrence. Spines were classified into Type 1-Fusiform shape, Type 2-Slender rod shape, Type 3-Thick rod shape, Type 4-Wooden club shape, and Type 5-Horizontal S-shape. Among the classified SS, Types 1 (47.17%) and 5 (19.18%) were the most common, followed Type 4 (13.21%) and 3 (12.58%), with Type 2 (7.86%) being the least common. The average length of landmarks AE, AC, and BC of the SS were 135.83 ± 10.33 mm, 83.27 ± 6.22 mm, and 45.60 ± 5.45 mm, respectively, which resemble previous published literature (133.6 ± 11.8 mm, 85.5 ± 8.7 mm, and 46.1 ± 6.3 mm, respectively).^14,15,26,27^

Type 5 SS classification obliquely crosses the dorsal surface of the scapula like a horizontal “S,” with a half forward cranial and the other half forward caudal. Although the other 4 types cross in a line from the vertical border to the scapular neck, the contour of Type 1 tappers at both ends with a wide middle region. Therefore, the contours of Types 5 and 1 reflect a more complex morphology than the other 4 types, eventually presenting a worse scenario with the presence of a fracture. It is an enormous challenge for the surgeon to bend and rotate the plate to fit the contour of these 2 types. Surgical time is delayed, there exists an increase in overlying tissue irritation, and it ends up aggravating the mechanics of the bone–plate construct.^30^ Furthermore, hardware removal rate is approximately 7.1% due to either implant-related discomfort or failure.^29,30^ Familiarizing with the morphological features may offer substantial benefits for the orthopedist in preoperative planning, and using precontoured locking plates may be an additional aid during surgery.

The SS has been usually regarded as an optimal region to support screw, pin, or wire purchase for fracture fixation stabilization because of the adequate bone stock.^14,17^ However, our study showed that the thickness of the landmarks of Types 2 and 5 were much thinner than those of types of Types 1, 3, and 4. Similarly, besides violent voluntary contraction of muscles, it has been believed that the fragility associated to the SS was one of the main reasons of avulsion fractures.^19–21^ Sanjay et al demonstrated high tensile and compressive stresses on the cranial and caudal sides of SSs, indicating high bending loads.^41^ This suggests that Types 2 and 5 might be more prone to fracture than other types. Furthermore, there exists a direct relationship between an increased screw pullout strength and the stability of the implant fixture with increased cortical thickness.^42,43^ As a result, it might be difficult to assess the fracture of the SS with internal fixation to a direct or indirect trauma associated to these 2 types.

Osteoporosis has been the only clinical factor to significantly increase the risk of scapular fractures after reverse shoulder arthroplasty.^11–13^ Although there are advantages with operative treatment, more recent research has recommended conservative management for the elderly patient with potentially osteopenic bone.^5,11^ Recently, it has been shown that the stability of the glenoid construct would be further enhanced by placing a longer posterior glenoid screw through the spinoglenoid notch and into the spine of the scapula.^44–46^ However, the study was limited by a significant variability in bone quality and size. Our study supplements this research by not recommending the addition of a longer posterior glenoid screw for Types 2 and 5, especially Type 2, because of the preexisting thinning spine. On the other hand, the bone stock and the thickness of the SS also have a substantial influence on the application of the osteomyocutaneous flap. Previous studies had demonstrated cortical thickness of donor bone to significantly affect stability of the fixture, known to be an important factor for osseointegration of the implant. Cancellous bone density is also an important factor related to a donor/recipient, biological response, but more importantly, to the mechanical support of the implant fixture.^42,43^ Although the average length and height of Types 2 and 5 were similar to other types, the preexisting thinning spine and limited bone stock including cortical and cancellous bone would be limiting factors in the osseointegration and with a subsequent reduction in support force.

The SS, as an osteomyocutaneous flap, has been previously used in the reconstruction of composite defects in the mandible.^31^ Studies have also expanded this method of reconstruction to other complex and variable defects, such as maxilla, pharyngeal, face, head, neck, humerus, and femur defects as well as trauma and congenital deformities.^32–36^ A study by Tubbs et al^38^ found the SS to be well-suited for posterior spinal fusion graft and successfully utilized it in posterior lumbar interbody fusion surgery. The SS has been versatile used in many areas of the body due to the ease of harvesting, minimal donor site morbidity as well as the reliable blood supply to this bone.^39,40^ An optimal osteomyocutaneous flap needs to be long and strong for bony union, and contoured to be able to reconstruct complex 3-dimensional skeletal defects.^32,33^ More importantly, estimating bone availability as well as familiarizing with the morphological features of the spine is essential for an appropriate contouring and fitting of the bone graft to the defects to ensure the best functional outcome.

There are several limitations to this study. First, although the SS has a very complex structure, the classification and measurements were carried out on dry specimens using a micrometer and caliper. More precise measurements could be obtained by analyzing a patient CT scan with possible 3D reconstruction models. However, these are costly and involve a significant amount of image analysis. Second, we report morphological measurements of 318 specimens of unknown sex and age that were collected from 1 university, preventing a comparison between genders and age differences. Third, as this is the first classification available on the SS according to morphological features on a Chinese population, we were unable to check the reliability and reproducibility of the classification types with other ethnic groups.

In conclusion, the present study classified and measured SS morphology on a large number Chinese specimens. Type 1 was the most common, while Type 2 was the least common. The contours of Types 5 and 1 were more complex than the other 3 types. Types 2 and 5 were much thinner than the other types; therefore, we believe these types to be more prone to fracture. The presented data provides precise and well-sorted information about SS variation and localization in a Chinese population. This supplements existing reports which contribute to a thorough understanding of the human SS.

## References

[1] Frankle M Siegal S Pupello D The Reverse Shoulder Prosthesis for glenohumeral arthritis associated with severe rotator cuff deficiency. A minimum two-year follow-up study of sixty patients. *J Bone Joint Surg Am* 2005; 87:1697–1705. 16085607 10.2106/JBJS.D.02813

[2] Werner CM Steinmann PA Gilbart M Treatment of painful pseudoparesis due to irreparable rotator cuff dysfunction with the Delta III reverse-ball-and-socket total shoulder prosthesis. *J Bone Joint Surg Am* 2005; 87:1476–1486. 15995114 10.2106/JBJS.D.02342

[3] Boileau P Watkinson DJ Hatzidakis AM Grammont reverse prosthesis: design, rationale, and biomechanics. *J Shoulder Elbow Surg* 2005; 14 (1 Suppl S):147S–161S. 15726075 10.1016/j.jse.2004.10.006

[4] Crosby LA Hamilton A Twiss T Scapula fractures after reverse total shoulder arthroplasty: classification and treatment. *Clin Orthop Relat Res* 2011; 469:2544–2549. 21448773 10.1007/s11999-011-1881-3 PMC3148370

[5] Hattrup SJ The influence of postoperative acromial and scapular spine fractures on the results of reverse shoulder arthroplasty. *Orthopedics* 2010; 33 5: 10.3928/01477447-20100329-04 20506958

[6] Walch G Mottier F Wall B Acromial insufficiency in reverse shoulder arthroplasties. *J Shoulder Elbow Surg* 2009; 18:495–502. 19250846 10.1016/j.jse.2008.12.002

[7] Cuff D Pupello D Virani N Reverse shoulder arthroplasty for the treatment of rotator cuff deficiency. *J Bone Joint Surg Am* 2008; 90:1244–1251. 18519317 10.2106/JBJS.G.00775

[8] Hamid N Connor PM Fleischli JF Acromial fracture after reverse shoulder arthroplasty. *Am J Orthop (Belle Mead NJ)* 2011; 40:E125–E129. 22013577

[9] Boileau P Watkinson D Hatzidakis AM Neer Award 2005: The Grammont reverse prosthesis: results in cuff tear arthritis, fracture sequelae, and revision arthroplasty. *J Shoulder Elbow Surg* 2006; 15:527–540. 16979046 10.1016/j.jse.2006.01.003

[10] Buquin T Hersan A Hubert L Reverse shoulder arthroplasty for the treatment of three- and four-part fractures of the proximal humerus in the elderly: a prospective review of 43 cases with a short-term follow-up. *J Bone Joint Surg Br* 2007; 89:516–520. 17463122 10.1302/0301-620X.89B4.18435

[11] Levy JC Anderson C Samson A Classification of postoperative acromial fractures following reverse shoulder arthroplasty. *J Bone Joint Surg Am* 2013; 95:e104. 23925750 10.2106/JBJS.K.01516

[12] Nicolay S De Beuckeleer L Stoffelen D Atraumatic bilateral scapular spine fracture several months after bilateral reverse total shoulder arthroplasty. *Skeletal Radiol* 2014; 43:699–702. 24276680 10.1007/s00256-013-1775-4

[13] Otto RJ Virani NA Levy JC Scapular fractures after reverse shoulder arthroplasty: evaluation of risk factors and the reliability of a proposed classification. *J Shoulder Elbow Surg* 2013; 22:1514–1521. 23659805 10.1016/j.jse.2013.02.007

[14] Burke CS Roberts CS Nyland JA Scapular thicknesses implications for fracture fixation. *J Shoulder Elbow Surg* 2006; 15:645–648. 16979064 10.1016/j.jse.2005.10.005

[15] von Schroeder HP Kuiper SD Botte MJ Osseous anatomy of the scapula. *Clin Orthop Relat Res* 2001; 131–139. 11210947 10.1097/00003086-200102000-00015

[16] DiStefano JG Park AY Nguyen TQ Optimal screw placement for base plate fixation in reverse total shoulder arthroplasty. *J Shoulder Elbow Surg* 2011; 20:467–476. 20926311 10.1016/j.jse.2010.06.001

[17] As-Sultany M Tambe A Clark DI Nonunion of a scapular spine fracture: Case report and management with open reduction, internal fixation, and bone graft. *Int J Shoulder Surg* 2008; 2:64–67. 20300318 10.4103/0973-6042.42202 PMC2840823

[18] Ogawa K Naniwa T Fractures of the acromion and the lateral scapular spine. *J Shoulder Elbow Surg* 1997; 6:544–548. 9437604 10.1016/s1058-2746(97)90087-2

[19] Morioka T Honma T Ogawa K Incomplete avulsion fractures of the scapular spine caused by violent muscle contraction. *Keio J Med* 2014; 63:13–17. 24334616 10.2302/kjm.2013-0004-cr

[20] García-Coiradas J Lópiz Y Marco F Stress fracture of the scapular spine associated with rotator cuff dysfunction: Report of 3 cases and review of the literature. *Rev Esp Cir Ortop Traumatol* 2014; 58:314–318. 24821480 10.1016/j.recot.2013.12.003

[21] Groot D Giesberts AM van Mourik JB Spontaneous scapular spine fracture related to rotator cuff pathology: a report of two cases. *Strategies Trauma Limb Reconstr* 2012; 7:105–107. 22610301 10.1007/s11751-012-0135-6 PMC3535129

[22] Goss TP Scapular fractures and dislocations: diagnosis and treatment. *J Am Acad Orthop Surg* 1995; 3:22–33. 10790650 10.5435/00124635-199501000-00004

[23] Armstrong CP Van der Spuy The fractured scapula: importance and management based on a series of 62 patients. *Injury* 1984; 15:324–329. 6706394 10.1016/0020-1383(84)90056-1

[24] Kuhn JE Blasier RB Carpenter JE Fractures of the acromion: a proposed classification system. *J Orthop Trauma* 1994; 8:6–13. 8169698 10.1097/00005131-199402000-00002

[25] Lollino N Caranzano F Paladini P Subacromial widening osteotomy of the scapular spine: surgical technique and literature review. *Injury* 2009; 40:1239–1241. 19232593 10.1016/j.injury.2008.10.018

[26] Ebraheim NA Xu R Haman SP Quantitative anatomy of the scapula. *Am J Orthop (Belle Mead NJ)* 2000; 29:287–292. 10784017

[27] Maves MD Philippsen LP Surgical anatomy of the scapular spine in the trapezius-osteomuscular flap. *Arch Otolaryngol Head Neck Surg* 1986; 112:173–175. 3942622 10.1001/archotol.1986.03780020053012

[28] Mallon WJ Brown HR Vogler JB 3rd Radiographic and geometric anatomy of the scapula. *Clin Orthop Relat Res* 1992; 277:142–154. 1555335

[29] Lantry JM Roberts CS Giannoudis PV Operative treatment of scapular fractures: a systematic review. *Injury* 2008; 39:271–283. 17919636 10.1016/j.injury.2007.06.018

[30] Park AY DiStefano JG Nguyen TQ Congruency of scapula locking plates: implications for implant design. *Am J Orthop (Belle Mead NJ)* 2012; 41:E53–E56. 22530212

[31] Panje W Cutting C Trapezius osteomyocutaneous island flap for reconstruction of the anterior floor of the mouth and the mandible. *Head Neck Surg* 1980; 3:66–71. 6997238 10.1002/hed.2890030112

[32] Chen WL Chen ZW Yang ZH The trapezius osteomyocutaneous island flap for reconstructing hemimandibular and oral defects following the ablation of advanced oral malignant tumours. *J Craniomaxillofac Surg* 2009; 37:91–95. 19062298 10.1016/j.jcms.2008.10.012

[33] Pinsolle V Tessier R Casoli V The pedicled vascularised scapular bone flap for proximal humerus reconstruction and short humeral stump lengthening. *J Plast Reconstr Aesthet Surg* 2007; 60:1019–1024. 17499036 10.1016/j.bjps.2007.03.028

[34] Bem C O’Hare PM Reconstruction of the mandible using the scapular spine pedicled upon trapezius muscle; description of the posterior approach to the transverse cervical vessels. *Br J Plast Surg* 1986; 39:473–477. 3779194 10.1016/0007-1226(86)90116-5

[35] Gregor RT Davidge-Pitts KJ Trapezius osteomyocutaneous flap for mandibular reconstruction. *Arch Otolaryngol* 1985; 111:198–203. 3977745 10.1001/archotol.1985.00800050092015

[36] Panje WR Mandible reconstruction with the trapezius osteomusculocutaneous flap. *Arch Otolaryngol* 1985; 111:223–229. 3883977 10.1001/archotol.1985.00800060047005

[37] Scapinelli R Posterior addition acromioplasty in the treatment of recurrent posterior instability of the shoulder. *J Shoulder Elbow Surg* 2006; 15:424–431. 16831645 10.1016/j.jse.2005.10.012

[38] Tubbs RS Wartmann CT Louis RG Jr Use of the scapular spine in lumbar fusion procedures: cadaveric feasibility study. Laboratory investigation. *J Neurosurg Spine* 2007; 7:554–557. 17977199 10.3171/SPI-07/11/557

[39] Vacher C de Vasconcellos JJ The anatomical basis of the osteo-musculo-cutaneous trapezius flap in mandibular reconstruction. *Surg Radiol Anat* 2005; 27:1–7. 15549301 10.1007/s00276-004-0278-3

[40] Hartman EH Spauwen PH Jansen JA Donor-site complications in vascularized bone flap surgery. *J Invest Surg* 2002; 15:185–197. 12217183 10.1080/08941930290085967

[41] Gupta S van der Helm FC Load transfer across the scapula during humeral abduction. *J Biomech* 2004; 37:1001–1009. 15165870 10.1016/j.jbiomech.2003.11.025

[42] Myoung H Kim YY Heo MS Comparative radiologic study of bone density and cortical thickness of donor bone used in mandibular reconstruction. *Oral Surg Oral Med Oral Pathol Oral Radiol Endod* 2001; 92:23–29. 11458242 10.1067/moe.2001.115027

[43] Niimi A Ozeki K Ueda M A comparative study of removal torque of endosseous implants in the fibula, iliac crest and scapula of cadavers: preliminary report. *Clin Oral Implants Res* 1997; 8:286–289. 9586475 10.1034/j.1600-0501.1997.080406.x

[44] Hoenig MP Loeffler B Brown S Reverse glenoid component fixation: is a posterior screw necessary? *J Shoulder Elbow Surg* 2010; 19:544–549. 20056452 10.1016/j.jse.2009.10.006

[45] Codsi MJ Iannotti JP The effect of screw position on the initial fixation of a reverse total shoulder prosthesis in a glenoid with a cavitary bone defect. *J Shoulder Elbow Surg* 2008; 17:479–486. 18282725 10.1016/j.jse.2007.09.002

[46] Klein SM Dunning P Mulieri P Effects of acquired glenoid bone defects on surgical technique and clinical outcomes in reverse shoulder arthroplasty. *J Bone Joint Surg Am* 2010; 92:1144–1154. 20439660 10.2106/JBJS.I.00778

